# Evidence for unconscious regulation of performance in fatigue

**DOI:** 10.1038/s41598-017-16439-6

**Published:** 2017-11-23

**Authors:** Akira Ishii, Masaaki Tanaka, Takahiro Yoshikawa, Yasuyoshi Watanabe

**Affiliations:** 1 Department of Sports Medicine, Osaka City University Graduate School of Medicine, 1-4-3 Asahimachi, Abeno-ku, Osaka, 545-8585 Japan; 2 Department of Physiology, Osaka City University Graduate School of Medicine, 1-4-3 Asahimachi, Abeno-ku, Osaka, 545-8585 Japan; 3 RIKEN, Center for Life Science Technologies, 6-7-3 Minatojima-minamimachi, Chuo-ku, Kobe, Hyogo, 650-0047 Japan

## Abstract

Since fatigue is prevalent in modern societies, it is necessary to clarify the neural mechanisms of fatigue. The regulation of performance through fatigue sensation is one of the mechanisms that decreases performance in fatigue. However, it is unknown whether subjective feeling of fatigue is necessary for the regulation of performance. Here, we examined whether decreased performance occurs without increased fatigue sensation through the experiment which was designed to test if fatigue can be learned unconsciously. Healthy male volunteers performed a fatigue-inducing hand-grip task for 10 min while viewing a target image presented without awareness. On the next day, they viewed a control and the target images presented with awareness and the neural activity caused by viewing the images was measured using magnetoencephalography. Results showed the level of fatigue sensation was not altered but grip-strength was decreased by viewing the target image on the second day. The level of beta band power in Brodmann’s area 31 was increased by viewing the target image and this increase was negatively associated with the decrease of grip-strength caused in the hand-grip task. These findings demonstrated that fatigue can be learned unconsciously and that there is a mechanism to decrease performance without fatigue sensation.

## Introduction

Fatigue is defined as a decline in the ability to perform or in the efficiency of performing mental and/or physical activities caused by excessive mental or physical activity or disease, and is often accompanied by a peculiar sense of discomfort, a desire to rest, and a decline in motivation; these feelings are referred to as fatigue sensation^1^. Fatigue is prevalent in modern societies^2–7^ and the complaint of fatigue is ubiquitous in primary care settings^8,9^. Fatigue is related to accidents and thus is a major contributor to workplace morbidity and mortality^10,11^. In addition, fatigue is associated with mortality in the general population especially through cardiovascular disease^12^. Therefore, clarifying the pathophysiology of fatigue to better deal with fatigue and its related problems is necessary.

As mentioned above, fatigue decreases the performance of activity. It is thought that this decrease in performance is not only due to the dysfunction of the part of the body and/or brain engaged in the activity, but is also due to the neural mechanisms which regulate the performance^13–18^. It has been proposed that two conceptual systems, the facilitation and inhibition systems, are involved in this mechanism. In the presence of workload, the facilitation system is activated through factors such as motivation to increase performance and, at the same time, the inhibition system is activated to decrease performance to maintain homeostasis. The balance between these two systems determines the performance of the activity to accomplish the workload^16,18^.

Fatigue sensation plays an important role as a biological alarm and urges us to rest to avoid disrupting homeostasis^19,20^. In our everyday lives, we sometimes limit performance to accomplish the assigned workload based on the subjective level of fatigue to avoid the accumulation of fatigue. It is important to decide whether or not to take a rest based on the subjective level of fatigue in order to prevent overwork, which can be a cause of death in the working population^21–23^. Thus, the regulation of performance through fatigue sensation is one of the mechanisms that decreases performance in the existence of workload. However, it is unclear whether awareness of the existence of fatigue, i.e., fatigue sensation, is necessary for suppressing performance. Since clarifying the neural mechanisms that decrease performance is central to understanding the pathophysiology of fatigue, it is worthwhile to investigate whether there are mechanisms that decrease performance independently of fatigue sensation.

Fear conditioning paradigms are often used to clarify the neural mechanism of the acquisition of fear response, in which the paired conditioned stimulus induces responses usually caused by an aversive unconditioned stimulus. In these fear conditioning paradigms, the stimuli are presented with the participant’s awareness (in awareness). However, there have been several reports that conditioned responses have been acquired when the conditioned stimuli were presented without the participant’s awareness (out of awareness), suggesting that associative learning can be acquired without awareness^24–29^. For example, it has been reported that a conditioned response was acquired with masked presentation of snakes and spiders associated with a mild electric shock as an unconditioned stimulus^25^, that associative learning between masked presentations of emotional facial expressions and shock stimuli occurred^30^, and that the associations between unconsciously presented words and a white noise blast as an unconditioned stimulus were observed^27^. In these studies, conditioned responses were assessed by measuring alterations in skin conductance response or electroencephalography (EEG). In a functional magnetic resonance imaging (fMRI) study, the effect of an unconsciously learned association between visual stimuli and an electrical unconditioned stimulus was shown by changes in neural activity, although conditioned subjective responses were not observed^29^. Taking this into consideration, there is a possibility that an association between fatigue and a stimulus presented out of awareness can be learned and, in addition, that a decrease in performance can be induced by the conditioned stimulus after the learning without an increase in fatigue sensation. If this happens, it is plausible that there is a mechanism that decreases performance independently of fatigue sensation.

In a previous magnetoencephalography (MEG) study, it was reported that the association between fatigue and a conditioned stimulus presented in awareness was learned. In that study, the participants performed hand-grip trials while listening to metronome sounds on the first day. Increases in fatigue sensation and sympathetic nerve activity were induced only by listening to the metronome sounds without hand-grip trials on the second day, and sympathetic nerve activity was associated with a decrease in the 8-13 Hz band power in the right dorsolateral prefrontal cortex^17^. Since the increase in sympathetic nerve activity and the decrease in parasympathetic nerve activity were thought to be related to fatigue^31–33^, the results show that the association between fatigue and the conditioned stimulus presented in awareness was learned.

In our present study, we hypothesized that there may be mechanisms which decrease performance without fatigue sensation and aimed to examine whether associative learning between fatigue and a visual stimulus presented out of awareness occurs and whether the alterations in the level of performance and/or fatigue sensation are induced by the visual stimulus presented in awareness after the learning. To this end, the experimental procedure of the previous study was modified to present a conditioned stimulus out of awareness: A visual backward masking procedure^34–38^ was used to present the conditioned stimulus out of awareness. Hand-grip trials were performed with unconscious presentations of the conditioned stimulus on the first day and changes in fatigue sensation, grip strength, autonomic activity, and oscillatory brain activity caused by viewing the conditioned stimulus presented in awareness were assessed on the second day. Presentation of a control image, which was not presented during hand-grip trials on the first day, was also conducted on the second day. We assessed the levels of perception of fatigue and performance fatigability related to the repetitive hand-grip trials^39^. We focused on: 1) whether the increase in fatigue sensation caused by viewing the conditioned stimulus on the second day was greater than that caused by viewing the control image on the second day, 2) whether grip strength was decreased by viewing the conditioned stimulus on the second day but not altered by viewing the control image on the second day, and 3) whether the neural activity caused by viewing the conditioned stimulus on the second day was different from that caused by viewing the control image on the second day. Since the neural activity related to learning of the association between fatigue and a conditioned stimulus presented in awareness has been successfully detected using MEG in previous studies^17,40^, we used MEG to assess the neural activity caused by viewing the conditioned stimulus and performed spatial filtering analyses of the MEG data to detect changes in oscillatory power that reflect changes in neural dynamics^41–43^.

## Methods

### Participants

Twenty healthy male volunteers aged 23.5 ± 3.0 years of age (mean ± standard deviation [SD]) participated in this study. All participants were right-handed according to the Edinburgh Handedness Inventory^44^. Current smokers, individuals with a history of mental illness, brain injury, or upper extremity disorder, and individuals taking chronic medications that affect the central nervous system were excluded. The participants were asked to refrain from caffeine or products containing caffeine for 12 h before each experimental day. The Ethics Committee of Osaka City University approved the study protocol (approval number, 3502). All participants provided written informed consent to participate in this study in accordance with the principles of the Declaration of Helsinki and the Ethical Guidelines for Medical and Health Research Involving Human Subjects in Japan (Ministry of Education, Culture, Sports, Science and Technology and Ministry of Health, Labor and Welfare).

### Experimental design

**Figure 1:**
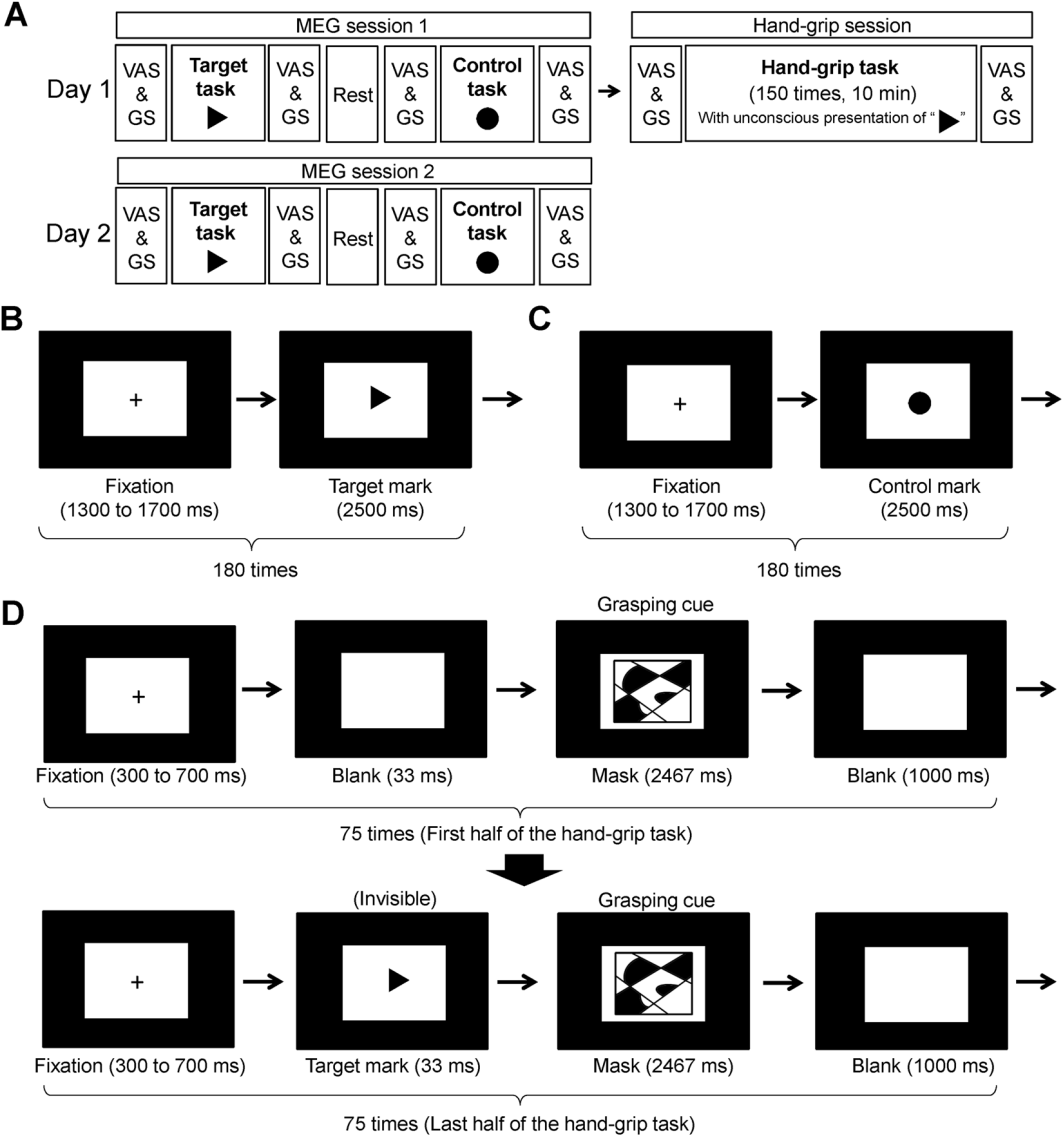
Experimental design. (**A**) The study was performed on two consecutive days. The experiment on the first day (day 1) consisted of an MEG session (MEG session 1), and a hand-grip session and that on the second day (day 2) consisted of an MEG session (MEG session 2) identical to that on day 1. In the MEG sessions, the participants were instructed to perform the target and control tasks. Just before and after the target, control, and hand-grip tasks, the participants were asked to rate their subjective level of fatigue using a visual analogue scale (VAS) and grip strength (GS) was assessed. At the end of the experiment on day 2, they were asked whether they recognized the existence of the target marks during the hand-grip task performed on day 1. (**B**) In the target task, they were asked to view an image, which was the repetition of a fixation cross for 1300 to 1700 ms followed by a target mark for 2500 ms (the target mark is a closed triangle in this figure). The target mark was presented 180 times. (**C**) In the control task, participants were asked to view another image, which was the repetition of a fixation cross for 1300 to 1700 ms followed by a control mark for 2500 ms (the control mark is a closed circle in this figure). The control mark was presented 180 times. There was a rest period of 5 min between the target and control tasks. (**D**) In the hand-grip session, they were asked to perform hand-grip trials for 150 times in time with a visual cue. The visual cue consisted of a fixation cross (for 300 to 700 ms), a blank screen or the target mark (for approximately 33 ms), a mask image as grasping cue (for approximately 2467 ms), and the blank screen (for 1000 ms). For the first half of the hand-grip trials (i.e., for 75 times), the blank screen was presented just before the mask image and the target mark was presented just before the mask image for the last half of the trials (i.e., for 75 times).

The study was performed on two consecutive days (Fig. 1). The experiment on the first day (day 1) consisted of an MEG session (MEG session 1), and a hand-grip session and that on the second day (day 2) consisted of an MEG session (MEG session 2) identical to that on day 1. In the MEG sessions, the participants lay on a bed placed in a magnetically shielded room and were instructed to perform the target and control tasks (Fig. 1A). In the target task, they were asked to view an image projected on a screen by a projector (PG-B10S; SHARP, Osaka, Japan). The image used in the target task was the repetition of a fixation cross for 1300 to 1700 ms followed by a target mark for 2500 ms (Fig. 1B; the target mark is a closed triangle in this figure). The target mark was presented 180 times during the target task. In the control task, participants were asked to view another image. This image used in the control task was the repetition of a fixation cross for 1300 to 1700 ms followed by a control mark for 2500 ms (Fig. 1C; the control mark is a closed circle in this figure). The control mark was presented 180 times during the control task. The target and control marks were the closed triangle and the closed circle, respectively, for half of the participants and the closed circle and the closed triangle, respectively, for another half of the participants. Whether the target mark was the closed triangle or the closed circle was randomly assigned for each participant. Magnetic neural activities over the target and control tasks were recorded using MEG. The order of the tasks (i.e., the target task followed by the control task or the control task followed by the target task on day 1 and 2) was randomized among the participants and there was a rest period of 5 min between the target and control tasks. On day 1, the participants performed the hand-grip session following the MEG session 1. In the hand-grip session, they were asked to perform hand-grip trials for 150 times with their right hand using a hand-grip device (EXG006 30 kg; ALINCO, Osaka, Japan) in time with a visual cue. The visual cue consisted of a fixation cross (for 300 to 700 ms), a blank screen or the target mark (for approximately 33 ms), a mask image as grasping cue (for approximately 2467 ms), and the blank screen (for 1000 ms) (Fig. 1D). This sequence of the visual presentation was used to make the target mark invisible for the participants during the hand-grip task trials (i.e., visual backward masking procedure). For the first half of the hand-grip trials (i.e., for 75 times), the blank screen was presented just before the mask image and the target mark was presented just before the mask image for the rest of the trials (i.e., for 75 times) so that the association between fatigue and the target mark could be learned in the last half of the hand-grip session. They were instructed to grasp the hand-grip device with their maximum power over the presentation of the mask image (i.e., all participants performed hand-grips 150 times). Just before and after the target, control, and hand-grip tasks, the participants were asked to rate their subjective level of fatigue using a 100-mm visual analogue scale (VAS) ranging from 0 (minimum) to 100 (maximum) and grip strength was assessed using a grip dynamometer (ST-100; Toei Light Co., Ltd., Saitama, Japan). At the end of the experiment on day 2, they were asked whether they recognized the existence of the target marks during the hand-grip task performed on day 1. The experiments for 10 participants were performed between 9 a.m. and noon and those for the others were performed between 2 p.m. and 5 p.m.

### MEG recording

MEG was recorded using a 160-channel whole-head-type MEG system (MEG vision; Yokogawa Electric Corporation, Tokyo, Japan) with a magnetic field resolution of 4 fT/Hz^1/2^ in the white-noise region. The sensor and reference coils were gradiometers with a 15.5-mm diameter and 50-mm baseline, and the two coils were separated by 23 mm. The sampling rate was 1000 Hz and data were high-pass filtered at 0.3 Hz.

### MEG analyses

The magnetic noise that originated from outside the magnetically shielded room was eliminated by subtracting the data obtained from reference coils using specialized software (MEG 160; Yokogawa Electric Corporation). Epochs of the raw MEG data that included artifacts were visually identified and were excluded before the analyses. Spatial filtering analysis of the MEG data was performed to identify changes in oscillatory brain activity that reflected time-locked cortical activities^41–43^ caused by performing the target and control tasks on day 2. The MEG data were bandpass filtered at 8–13 Hz and 13–25 Hz by a finite impulse response filtering method using Brain Rhythmic Analysis for MEG software (BRAM; Yokogawa Electric Corporation) to obtain alpha and beta signals, respectively. After the bandpass filtering, the location and intensity of the cortical activities were estimated using BRAM, which uses a narrow-band adaptive spatial filtering algorithm^45,46^. Voxel size was set at 5.0 × 5.0 × 5.0 mm. For each MEG measurement (i.e., both the target and control tasks) and frequency band, the oscillatory power of the MEG data from 0 to 2000 ms after the onset of the target or control marks was calculated relative to that from −500 to 0 ms before the onset of the marks. Data were then analyzed using statistical parametric mapping (SPM8; Wellcome Department of Cognitive Neurology, London, UK), implemented in Matlab (Mathworks, Natick, MA). The MEG parameters were transformed into the Montreal Neurological Institute T1-weighed image template^47^ and applied to the MEG data. The anatomically normalized MEG data were filtered with a Gaussian kernel of 20 mm (full-width at half-maximum) in the x-, y-, and z-axes. Individual data were summarized and incorporated into a random-effect model^48^. The weighted sum of the parameters estimated in the individual analyses was used to create “contrast” images that were used for group analyses^48^. The resulting set of voxel values for each comparison constituted a statistical parametric map (SPM) of the *t* statistic (SPM{*t*}). The SPM{*t*} was transformed to units of normal distribution (SPM{Z}). The significance of any changes in the oscillatory power observed in the target and control tasks and the significance of any difference in the oscillatory power observed during the target task compared to that observed during the control task were assessed using *t* statistics (one sample *t*-test and paired *t*-test, respectively) on a voxel-by-voxel basis^48^. The threshold for the SPM{*t*} of the one sample *t*-test and paired *t*-test was set at *P* < 0.05 (family-wise-error corrected for multiple comparisons). Localization of the brain regions was performed using WFU_PickAtras, Version 3.0.4 (http://fmri.wfubmc.edu/software/pickatlas) and Talairach Client, Version 2.4.3 (http://www.talairach.org/client.html).

### Magnetic resonance (MR) image overlay

Anatomical MR imaging was performed using a Philips Achieva 3.0 TX (Royal Philips Electronics, Eindhoven, The Netherlands) to permit registration of magnetic source locations with their respective anatomical locations. Before MR scanning, five adhesive markers (Medtronic Surgical Navigation Technologies Inc., Broomfield, CO) were attached to the skin of the head: Two markers 10 mm in front of the left and right tragus, one marker 35 mm above the nasion, and two markers 40 mm to either side of the marker above the nasion. The MEG data were superimposed on MR images using information obtained from these markers and MEG localization coils.

### Electrocardiography (ECG)

To examine changes in autonomic nerve activity caused by performing the target and control tasks, ECG was recorded during the MEG recordings. The ECG data were analyzed with the maximum entropy method using MemCalc for Windows (Global Medical Solution Inc., Tokyo, Japan). The ECG data from 300 to 600 s after the onset of the MEG recordings were used. R-R wave variability was measured as an indicator of autonomic nerve activity. For frequency-domain analysis of the R-R wave intervals, low-frequency (LF) power was calculated as the power within the frequency range 0.04–0.15 Hz and high-frequency (HF) power was calculated as the power within the frequency range 0.15–0.4 Hz. LF and HF power were measured in absolute units (ms^2^). It has been reported that HF power is vagally mediated^49–51^, but that LF power originates from a variety of sympathetic and vagal mechanisms^49,52^. The LF/HF ratio is considered an index of sympathetic nervous system activity^53^. The natural logarithm of LF power, HF power, and the LF/HF ratio was calculated and used for statistical analyses.

### Statistical analyses

A paired *t*-test was used to compare the subjective level of fatigue, grip strength, and indices of autonomic nerve function between the target and control tasks and the subjective level of fatigue and grip strength between before and after the hand-grip task. Pearson’s correlation analysis was conducted to evaluate the relationships between the MEG responses induced by the target task on day 2 and the change in grip strength caused by the hand-grip task. All *P* values were two-tailed and values less than 0.05 were considered statistically significant. All statistical analyses were performed using the IBM SPSS 21.0 software package (IBM, Armonk, NY) and values are presented as mean and SD unless otherwise stated.

## Results

### Awareness of the existence of the target mark during the hand-grip task

Three participants noticed the existence of the target marks during the hand-grip task performed on day 1. Two participants reported that they saw the target marks several times during the hand-grip task and one participant reported that he saw the target marks just before the appearance of the mask image almost every time the mask image appeared. The data from these three participants were excluded from our analyses. The others declared that they did not recognize the target marks at all during the hand-grip task. In addition, the MEG data from the other two participants were also excluded from the analyses because their MEG data were contaminated with magnetic noise that originated from outside the shielded room and the number of epochs that remained after exclusion of the epochs that included artifacts was not sufficient for the analysis: We used the MEG data with more than 10 epochs for the analyses. Therefore, the final sample size of our present study was 15.

### Subjective level of fatigue

**Figure 2:**
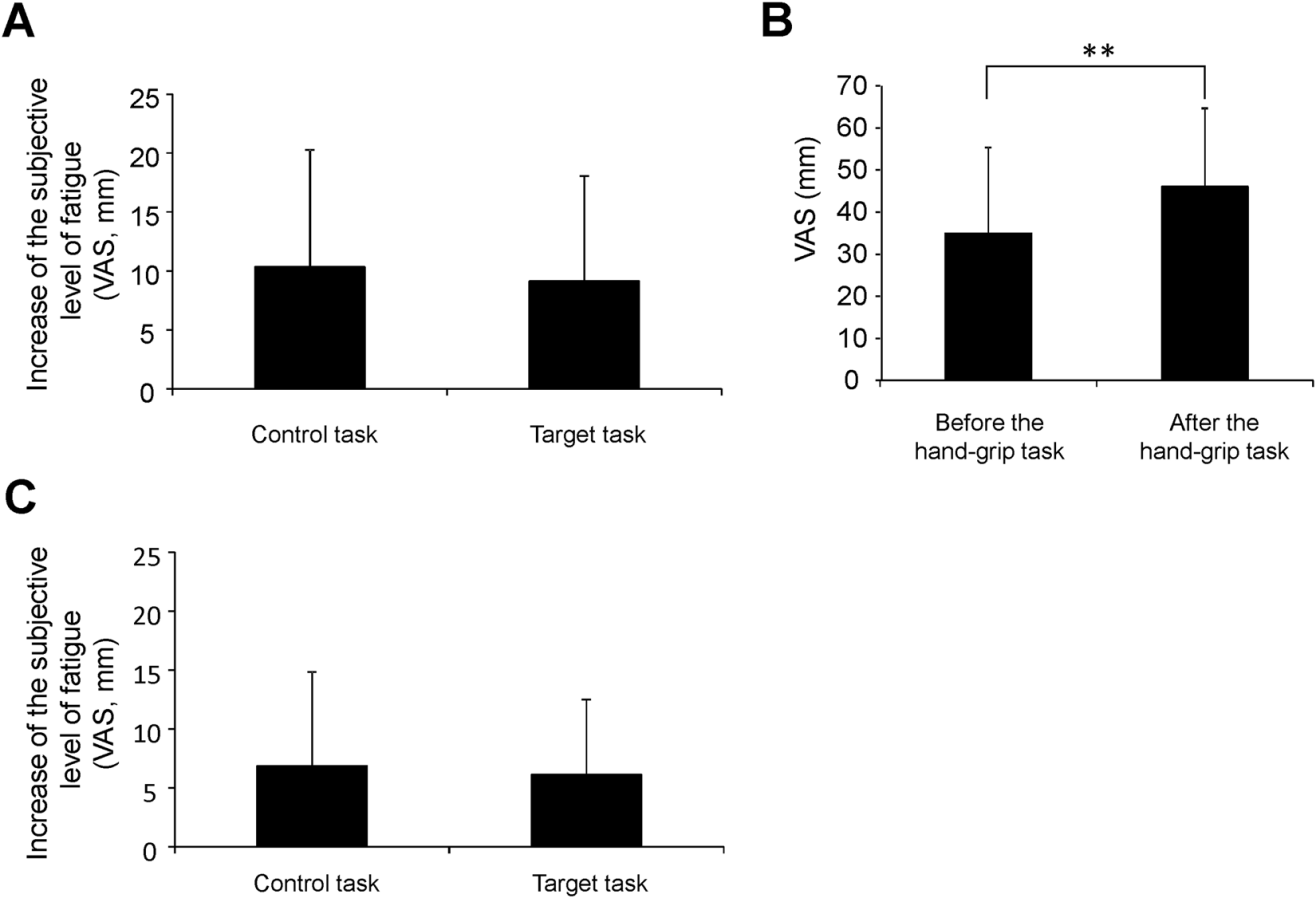
Subjective level of fatigue. The increases in the subjective level of fatigue during the control and target tasks in MEG session 1 (**A**), the levels of fatigue sensation before and after the hand-grip session (**B**), and the increases in the subjective level of fatigue during the control and target tasks in MEG session 2 (**C**) were shown. Participants were asked to rate their subjective level of fatigue on a 100-mm visual analogue scale (VAS) ranging from 0 (minimum fatigue) to 100 (maximum fatigue). Data are presented as means and standard deviations. **P < 0.01, paired *t*-test.

We were interested in whether the increase in the subjective level of fatigue caused by the target task on day 2 was different from that caused by the control task on day 2. The increase in the subjective level of fatigue observed during the target task on day 2 was not altered compared with that observed during the control task on day 2 (*t*_14_ = 0.426; *P* = 0.677, paired *t*-test; Fig. 2C). The subjective level of fatigue observed after the hand-grip task on day 1 was increased compared with that before the task (*t*_14_ = 0.5.385; *P* < 0.001, paired t-test; Fig. 2B).

### Grip strength

**Figure 3:**
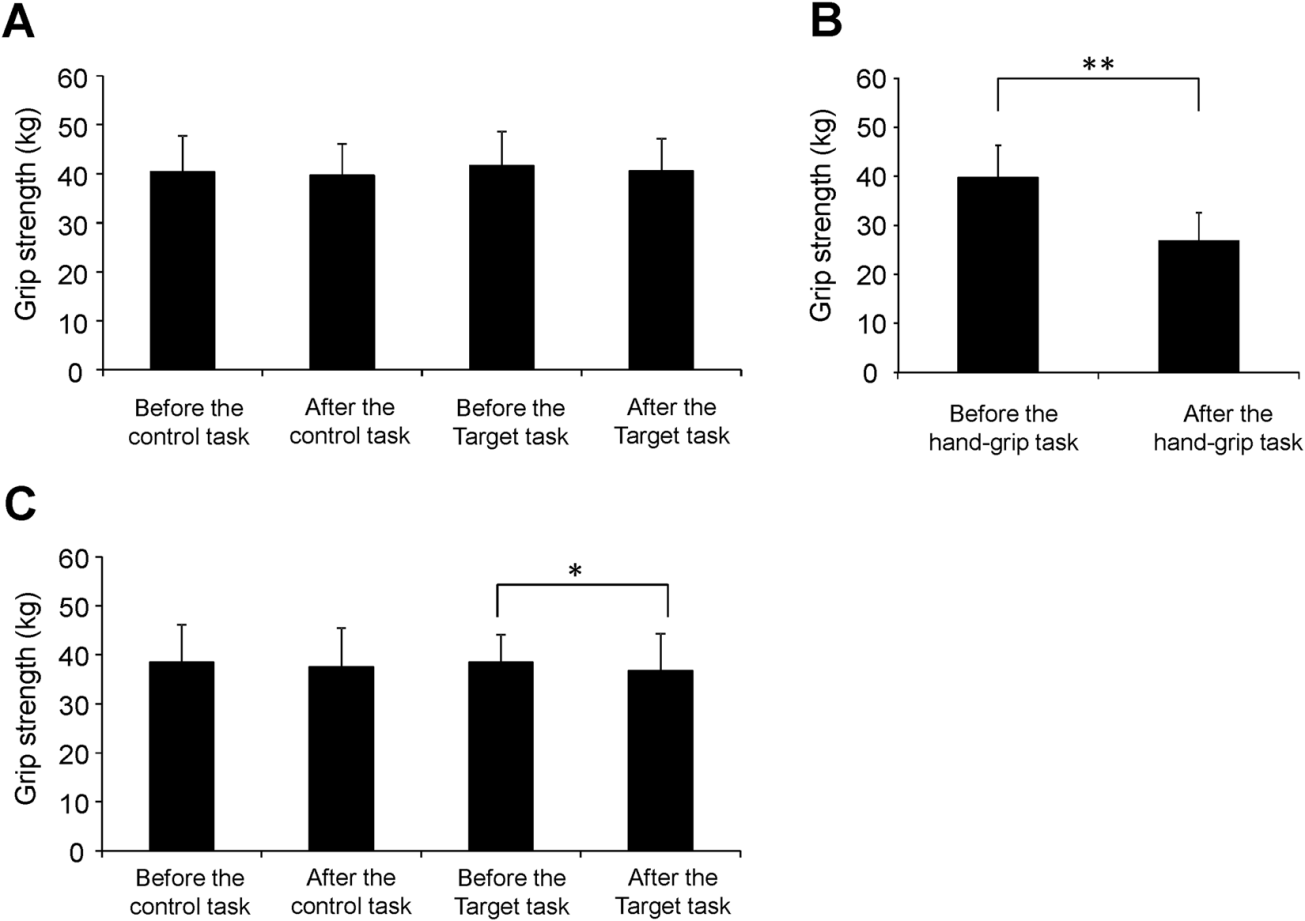
Grip strength. Grip strength assessed before and after the control and target tasks in MEG session 1 (**A**), that assessed before and after the hand-grip task (**B**), and that assessed before and after the control and target tasks in MEG session 2 (**C**) were shown. Data are presented as means and standard deviations. ^*^ *P* < 0.05 and ^**^ *P* < 0.01, paired *t*-test.

We were interested in whether grip strength was decreased by performing the target task on day 2 while remaining the same during the control task on day 2. The grip strength assessed just after the target task on day 2 was decreased compared with that assessed just before the target task on day 2 (*t*_14_ = 2.313; *P* = 0.036, paired *t*-test; Fig. 3C) and the grip strength assessed just after the control task on day 2 was not altered compared with that assessed just before the control task on day 2 (*t*_14_ = 1.421; *P* = 0.177, paired t-test; Fig. 3C). The grip strength assessed just after the hand-grip task on day 1 was decreased compared with that assessed just before the hand-grip task on day 1 (*t*_14_ = 8.392; *P* < 0.001, paired t-test; Fig. 3B).

### Autonomic nerve activity

**Figure 4:**
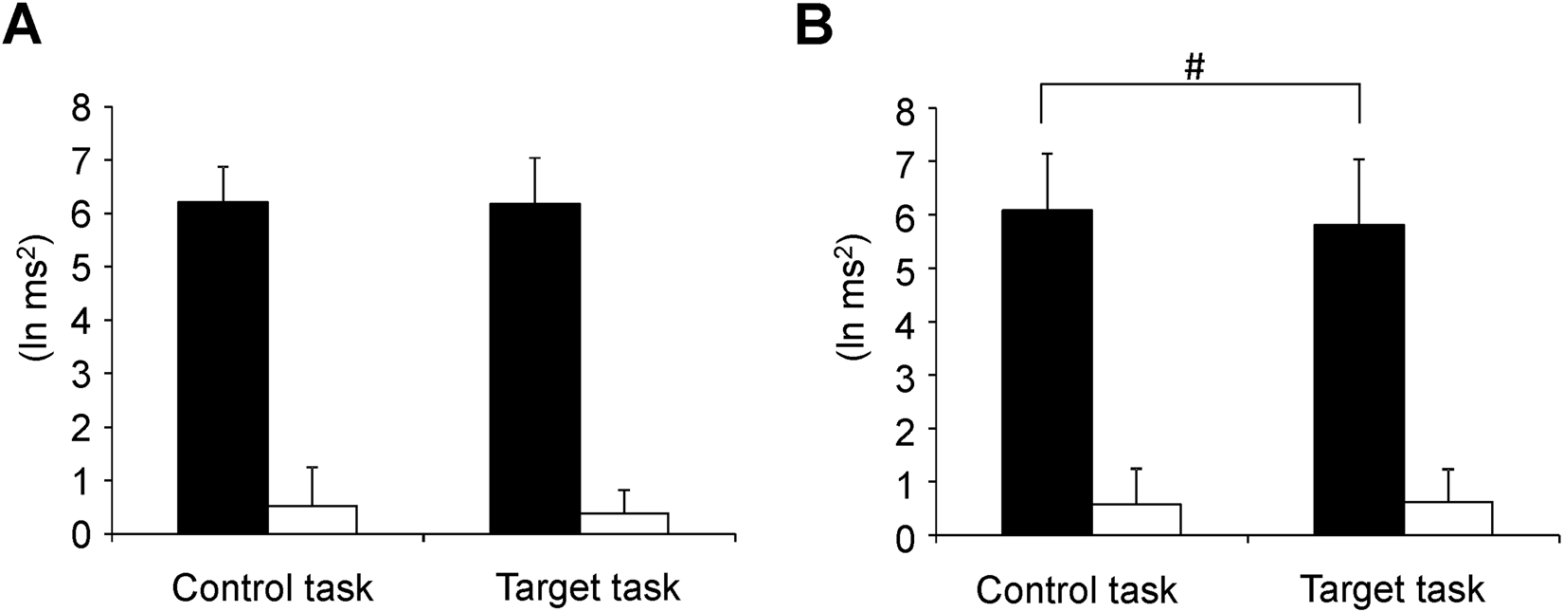
Autonomic nerve activity evaluated using frequency domain analysis of R-R wave intervals measured with electrocardiography. Values were transformed by natural logarithm (ln). High frequency power (ln HF, closed columns) and the LF/HF ratio (ln LF/HF; open columns) on day 1 (**A**) and day 2 (**B**) are shown. Data are presented as means and standard deviations. ^*#*^ *P* < 0.10, paired *t*-test with Bonferroni correction.

HF power and the LF/HF ratio were compared between the target and control tasks on day 2. The HF power observed during the target task on day 2 showed a tendency toward decrease compared with that observed during the control task on day 2 (*t*_14_ = 2.269; *P* < 0.079, paired *t*-tests with Bonferroni correction; Fig. 4B).

### Spatial filtering analyses of MEG data

**Figure 5:**
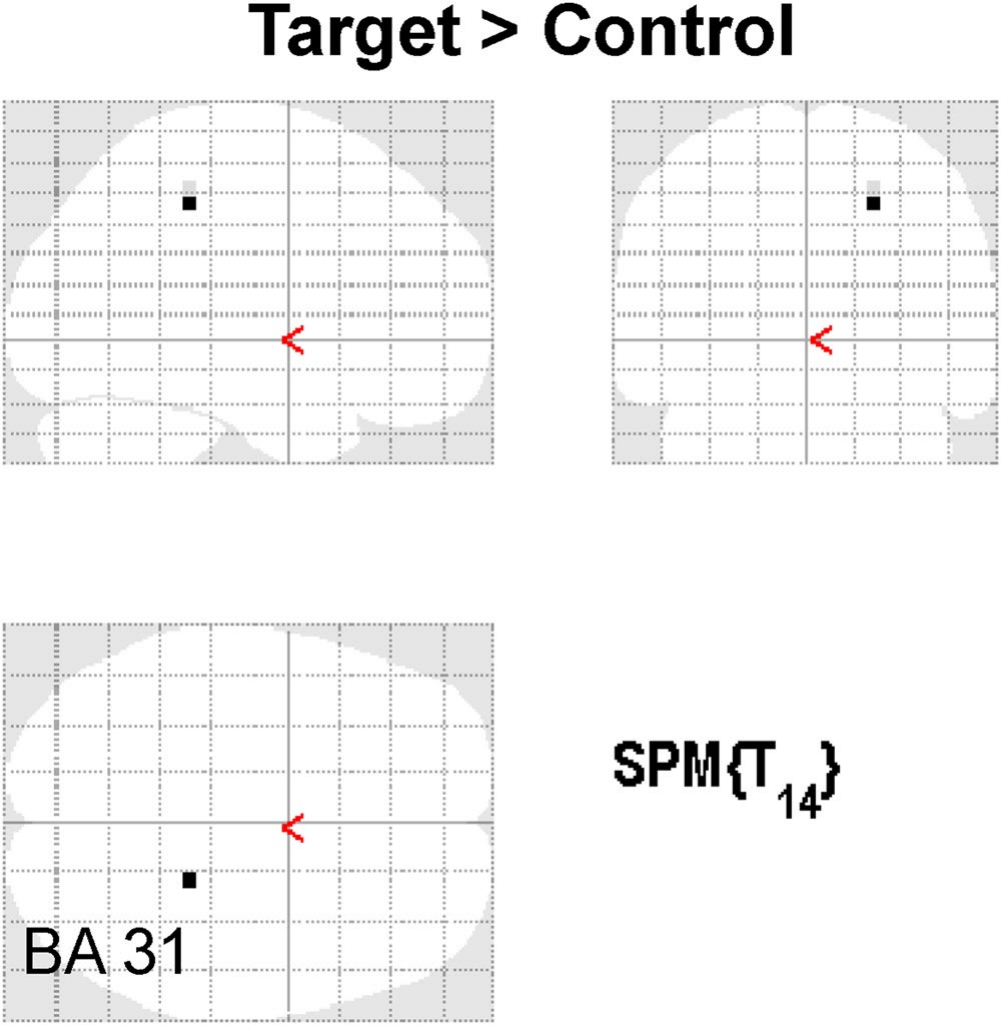
Statistical parametric map. Statistical parametric map of the brain region where the level of increased beta band (13–25 Hz) power was higher in the target task than that in the control task on day 2 is shown (maximum intensity projection). Random-effect analyses of 15 participants, *P* < 0.05, family-wise-error corrected for the entire search volume.

**Figure 6:**
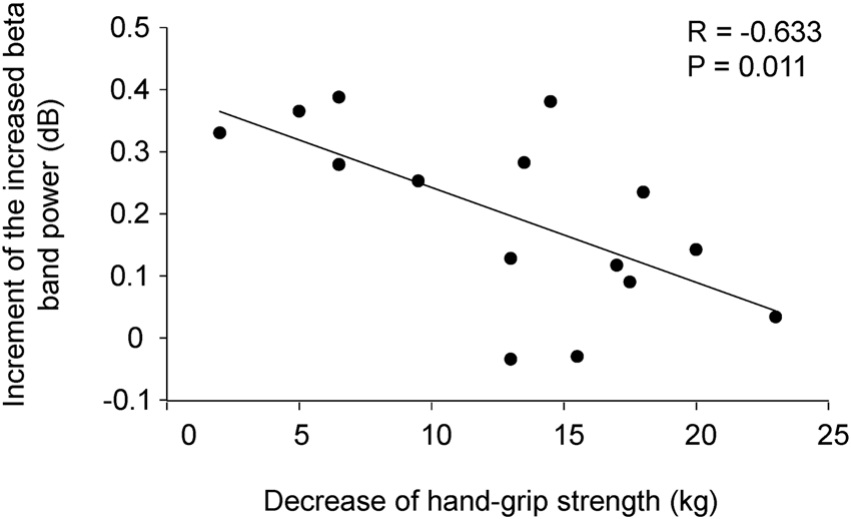
Relationship between the decrease in grip strength and the increment of increased beta (13–25 Hz) band power. Relationship between the decrease in grip strength induced by the hand-grip task on day 1 and the increment of increased beta band power in the target task compared with the control task on day 2 is shown. The linear regression line, Pearson’s correlation coefficient, and *P* value are shown.

Some brain regions showed changes in oscillatory powers during performing the target or control tasks (Table 1). To identify changes in neural activity caused by performing the target task compared with those caused by performing the control task on day 2, the oscillatory powers observed during the target task on day 2 were compared with those observed in the control task on day 2. There was one brain region in which the increase in the beta band power caused by performing the target task was larger than that caused by performing the control task (Table 2, Fig. 5). This brain region was located in the right posterior cingulate gyrus (Brodmann’s area 31; BA 31). The increased beta band power observed during the target task compared with that observed during the control task on day 2 was negatively associated with a decrease in grip strength caused by performing the hand-grip task on day 1 (R = −0.633, *P* = 0.011; Fig. 6).

**Table 1:** Brain regions that showed a decrease or increase in oscillatory power in the target or control tasks on day 1 and 2.

Experimental day	Task	Decrease/Increase	Frequency	Location	BA	MNI coordinate (mm)			Z value
						x	y	z	
Day 1	Control	Increase	8–13 Hz	Fusiform Gyrus	19	37	−77	−20	5.27
				Superior Temporal Gyrus	22	67	−27	5	5.09
				Middle Frontal Gyrus	6	22	−12	60	4.98
			13–25 Hz	Middle Temporal Gyrus	21	−53	8	−20	4.82
				Inferior Occipital Gyrus	18	47	−87	−15	4.70
				Middle Temporal Gyrus	39	42	−72	25	4.69
	Target	Increase	13–25 Hz	Middle Occipital Gyrus	19	−48	−72	5	3.71
				Fusiform Gyrus	19	−33	−82	−20	3.63
				Fusiform Gyrus	20	57	−17	−30	3.55
				Inferior Parietal Lobule	40	57	−42	45	3.07
Day 2	Control	Decrease	13–25 Hz	Postcentral Gyrus	3	22	−37	70	4.16
		Increase	8–13 Hz	Lingual Gyrus	18	7	−92	−20	4.16
				Lingual Gyrus	18	−3	−92	−20	4.15
				Middle Frontal Gyrus	6	37	3	55	4.13
				Precuneus	7	12	−82	45	2.97
			13–25 Hz	Cuneus	18	−23	−97	−5	3.48
				Superior Frontal Gyrus	9	−3	53	35	3.47
				Superior Frontal Gyrus	9	7	53	35	3.37
				Superior Frontal Gyrus	8	−18	33	55	3.02
				Uncus	20	−28	3	−50	3.26
				Middle Temporal Gyrus	38	−43	8	−40	3.23
				Superior Temporal Gyrus	38	−53	18	−30	3.23
				Precentral Gyrus	6	−28	−22	70	3.06
				Middle Temporal Gyrus	21	−68	−37	−5	3.04
				Middle Temporal Gyrus	21	−63	−42	−10	2.91
				Inferior Temporal Gyrus	21	67	−12	−20	3.04
				Parahippocampal Gyrus	36	27	−17	−30	3.00
				Superior Temporal Gyrus	22	67	−7	5	2.93
				Middle Temporal Gyrus	39	47	−77	20	2.87
	Target	Decrease	13–25 Hz	Lingual Gyrus	18	17	−72	−5	4.06
		Increase	8–13 Hz	Superior Temporal Gyrus	38	32	18	−45	4.12
				Middle Frontal Gyrus	6	32	18	60	4.06
				Superior Temporal Gyrus	38	32	8	−50	3.95
			13–25 Hz	Inferior Frontal Gyrus	47	−13	33	−15	4.22
				Uncus	28	−18	8	−30	3.70
				Inferior Frontal Gyrus	9	57	13	30	3.65
				Postcentral Gyrus	3	−43	−27	65	3.20
				Inferior Parietal Lobule	40	−58	−47	45	3.12
				Precentral Gyrus	4	−33	−27	70	3.06
				Superior Frontal Gyrus	6	−13	3	75	3.01
				Middle Frontal Gyrus	6	−28	8	65	3.00
				Superior Frontal Gyrus	8	−38	18	55	3.00

BA, Brodmann area; MNI, Montreal Neurological Institute.

x, y, z: Stereotaxic coordinate.

Data were obtained from random-effect analyses. Only significant changes are shown (one-sample *t* test, *P* < 0.05, family-wise error rate).

**Table 2:** The brain region that showed a change in oscillatory power in the target task compared with that in the control task.

Experimental day	Decrease/Increase	Frequency	Location	BA	MNI coordinate (mm)			Z value
					x	y	z	
Day 2	Increase	13–25 Hz	Cingulate Gyrus	31	22	−37	45	3.83

BA, Brodmann area; MNI, Montreal Neurological Institute.

x, y, z: Stereotaxic coordinate.

Data were obtained from random-effect analyses. Only significant changes are shown (paired *t* test, *P* < 0.05, family-wise error rate).

## Discussion

In the present study, the participants viewed masked target marks during a hand-grip task on day 1. A decrease in hand-grip strength and an increase in fatigue sensation were induced by the hand-grip trials, suggesting that fatigue and fatigue sensation were induced by the hand-grip trials. On day 2, an increase in fatigue sensation caused by viewing the target marks presented in awareness was not greater than that caused by viewing the control marks presented in awareness, and a decrease in grip strength was induced by viewing the target marks while a decrease in grip strength was not induced by viewing the control marks. In addition, the level of increased beta band power in BA 31 was greater while viewing the target marks than that while viewing the control marks on day 2, and an increase in the beta band power observed during the target task was negatively associated with a decrease in grip strength caused by performing the hand-grip task on day 1.

On day 1, the target marks were presented out of awareness using a visual backward masking procedure^34–38^. Since three participants reported that they could recognize the masked target marks during the hand-grip task, the data from these participants were excluded from the analyses. The other participants, whose data were included in our analyses, were completely unaware of the presentation of the masked target marks on day 1.

The increased fatigue sensation caused by viewing the target marks presented in awareness was not greater than that caused by viewing the control marks presented in awareness on day 2. We often experience that participants’ subjective ratings of fatigue sensation increase while they are performing a task even if the load of the task is not high, such as only viewing simple images as in our present study. Therefore, we assessed whether there was difference between the increment of fatigue sensation caused by viewing the target mark and that caused by viewing the control mark on day 2. As a result, the increment of the fatigue sensation caused by viewing the target mark was not altered compared with that caused by viewing the control mark on day 2, showing that there were no significant effects of viewing the target mark on the level of fatigue sensation on day 2.

A decrease in grip strength was not observed in the control task but was observed in the target task on day 2. Since it is not plausible that grip strength is decreased only by viewing a “triangle” or “circle”, which have not been associated with fatigue, we examined whether grip strength was decreased by viewing the target mark but remained the same when viewing the control mark. Our findings regarding grip strength can be interpreted as follows: The association between the neural activity which reduced performance in the hand-grip task session and the target mark was learned out of awareness on day 1, resulting in the decrease in performance observed in the target task on day 2. In addition, taking into consideration the level of fatigue sensation was not altered by the target task on day 2, fatigue sensation is not absolutely necessary for suppressing performance.

Since we confirmed that the participants were unaware of the target mark in the hand-grip task, that the decrease in performance was induced by viewing the target mark on day 2, and that the increase in fatigue sensation was not induced by viewing the target mark on day 2, as we hypothesized in the Introduction, it was demonstrated that the association between fatigue and a stimulus presented out of awareness can be learned and that there is a mechanism that decreases performance independently of fatigue sensation.

It has been reported that an increase in sympathetic nerve activity and a decrease in parasympathetic nerve activity are related to fatigue. A decrease in HF power and an increase in the LF/HF ratio were observed in relation to the fatigue induced by performing mental fatigue-inducing tasks^31–33^ and an increase in the LF/HF ratio was observed after the induction of fatigue through a classical conditioning procedure^54^. Therefore, we assessed the changes of HF power and the LF/HF ratio caused by the target task on day 2 and, in fact, HF power over the target task showed a tendency toward decrease compared with that over the control task on day 2. This result may suggest that the alteration in autonomic nerve activity caused by fatigue was also learned out of awareness during the hand-grip task on day 1.

In addition to changes in fatigue sensation and grip strength, we assessed changes in the neural activity caused by viewing the target mark to examine whether fatigue can be learned out of awareness. There have been several reports that the alterations in oscillatory brain activity, especially alpha (8–13 Hz) and beta (13–25 Hz) band powers, are related to fatigue. In an EEG study, the beta power density on the Pz electrode and the alpha power density on the P3 and O2 electrodes were decreased by performing a fatigue-inducing two-back task for 30 min^55^. In MEG studies, the increase in beta band power in the frontal brain region was associated with the levels of boredom and sleepiness induced by fatigue^56^ and alterations in alpha band power in the dorsolateral prefrontal cortex were observed in relation to the regulation of performance in fatigue^15,17^. Thus, we examined alterations in oscillatory brain activity in the alpha and beta band powers caused by the target task. Our findings that the extent of the increased beta band power in BA 31 caused by viewing the target mark was greater than that caused by viewing the control mark, and that the increase in the beta band power was associated with the decrease in grip strength observed in the hand-grip task clearly demonstrate that fatigue was learned unconsciously on day 1. BA 31 is a brain region reported to be involved in the self-evaluation of the level of fatigue^57,58^, the classical conditioning of fatigue^17,40^, and the neural mechanisms of deciding to rest in the presence of fatigue^23^ and seems be related to the regulation of performance in fatigue. Taking that the increase in the beta band power was related to the levels of boredom and sleepiness induced by fatigue^59^, one possible explanation of our results is that the increase in the beta band power in BA 31 reflected the activation of the inhibition system caused by the hand-grip task^16,18^. The participants whose grip strength readily reduced in the hand-grip trials needed no further activation of the inhibition system on day 1. However, an alternative explanation that the increase in the beta band power in BA 31 reflected the deactivation of the facilitation system is also possible; the participants whose grip strength readily reduced in the hand-grip task trials needed no further deactivation of the facilitation system on day 1. Since it is difficult to determine whether the increase in the beta band power was related to the activation of the inhibition system, the deactivation of the facilitation system, or both in our present study, further study is needed on this point.

Since it is thought that motivation is related to the facilitation system of fatigue and that boredom and sleepiness are related to the inhibition system of fatigue^16,18,57^, motivation and arousal are the important factors which affect task performance. Therefore, the increase of the subjective level of fatigue (i.e., the level of perception of fatigue) and the decrease of the grip strength induced by the hand-grip task trials observed on day 1 would have been related to the alterations in the motivation and/or arousal caused in the hand-grip session on day 1. However, it seems that the decreases in motivation and/or arousal were not caused in the target task on day 2. It has been reported that the increase of motivation is associated with the increased activation of the sympathetic nerve system^60,61^ and that the reduced arousal is related to the increased activation of the parasympathetic nerve system^62,63^. In our present study, while the index of sympathetic nerve activity (i.e., LF/HF ratio) was not altered between the target and control tasks on the second day, the index of parasympathetic nerve activity (i.e., HF power) in the target task showed tendency toward decrease compared with that in the control task on day 2, suggesting that the activity of the parasympathetic nerve system was not increased in the target task. Taking these into consideration, the decreases of the level of motivation and/or arousal seem not to be caused in the target task on day 2 through our experimental procedure.

There are limitations to our study. First, the neural activity over the hand-grip task was not recorded in our present study because performing the hand-grip trials causes electromagnetic noise that disables the measurement of MEG. However, it is of great interest to directly investigate the neural mechanisms activated during the unconscious learning of fatigue. Second, all participants in our present study were healthy males. To generalize our results and to clarify the pathophysiology of fatigue, studies with healthy females or participants suffering from fatigue or fatigue related diseases would be of great value.

In conclusion, based on the behavioral and neural findings such as the alterations in the level of fatigue sensation, grip strength, and oscillatory brain activities assessed by MEG, we demonstrated that fatigue can be learned unconsciously and that there is a mechanism to decrease performance independently of fatigue sensation. Our findings will greatly contribute to a better understanding of the neural mechanisms that decrease performance under the condition of fatigue and provide clues to prevent and deal with fatigue and fatigue related problems.
